# Accidental cannabis intoxication in two young children: clinical presentation and toxicokinetics - a case series

**DOI:** 10.3389/fphar.2025.1695194

**Published:** 2025-11-11

**Authors:** Alessia Cafaro, Federica Pigliasco, Sebastiano Barco, Ilaria Negro, Emanuela Piccotti, Luca Manfredini, Samir Mahameed, Roberto Bandettini, Carla Debbia, Francesca Mattioli, Giuliana Cangemi

**Affiliations:** 1 Biochemistry, Pharmacology and Newborn Screening Unit, Central Laboratory of Analysis, IRCCS Istituto Giannina Gaslini, Genoa, Italy; 2 Pediatric Emergency Room and Emergency Medicine, IRCCS Istituto Giannina Gaslini, Genoa, Italy; 3 Pediatric Pain and Palliative Care Service, IRCCS Istituto Giannina Gaslini, Genoa, Italy; 4 Department of Internal Medicine, Pharmacology & Toxicology Unit, University of Genoa, Genoa, Italy; 5 Clinical Pharmacology Unit, Ente Ospedaliero Ospedali Galliera, Genoa, Italy

**Keywords:** cannabis, intoxication, children, pharmacokinetics, toxicokinetic

## Abstract

Accidental exposure to edible cannabis products in children is an increasing public health concern. The clinical presentation is often nonspecific, which can delay diagnosis and lead to inappropriate management. Toxicological screening is therefore essential for accurate diagnosis and appropriate treatment. We report the toxicokinetic (TK) profiles of delta-9-tetrahydrocannabinol (THC) and cannabidiol (CBD) following unintentional ingestion of cannabis in two pediatric cases, aged 12 and 15 months, respectively. Plasma concentrations of THC and CBD were measured using a validated liquid chromatography–tandem mass spectrometry (LC-MS/MS) method. THC was consistently detected in all plasma samples collected (four per patient), with highest measured concentrations of 45.0 μg/L in Case 1 and 54.7 μg/L in Case 2. CBD was not detected in Case 1, whereas in Case 2 it was measurable only in the first plasma sample, at a concentration of 1.11 μg/L. Non-compartmental analysis (NCA) of the THC concentration–time data enabled calculation of the TK profiles in both cases. The elimination rate constant (k_el_) was 0.013 h^−1^ in Case 1 and 0.031 h^−1^ in Case 2, corresponding to an half-life (t½) of 52.5 and 21.7 h, respectively. Given the variability and unpredictability of THC/CBD TK monitoring drug levels over time is crucial for managing intoxications in children.

## Introduction

The escalating accidental exposure of children to edible Cannabis (Cannabaceae family) products and passive smoke is becoming a significant concern (Richards et al., 2017; Vo et al., 2018). This issue includes instances of passive smoke inhalation, ingestion of various products made from different species of Cannabis plants, such as cannabis-infusions or cookie-like preparations, and the availability of unprotected or partially consumed hashish or joints (Richards et al., 2017). In many cases, ingestion occurs when young children encounter and consume cannabis-containing products (CCPs) that have been left unattended by adults (Richards et al., 2017; Vo et al., 2018). This type of exposure is often described as “exploratory,” as children naturally investigate unfamiliar objects by mouthing or ingesting them (Richards et al., 2017). Ingestion represents the most common route of intoxication in pediatric cases, with symptom onset typically occurring within 1.5–3.5 h, although delays up to 4–6 h have been reported. The most frequently observed clinical signs in children exposed to CCPs include lethargy, ataxia, hypotonia, mydriasis, tachycardia, and hypoventilation (Richards et al., 2017; Morini et al., 2018; Spadari et al., 2009; Gaulier et al., 2002; Carstairs et al., 2011; Macnab et al., 1989). These symptoms often persist for a prolonged duration, ranging from 6 to 8 h up to 24 h (Richards et al., 2017; Morini et al., 2018; Spadari et al., 2009; Gaulier et al., 2002; Carstairs et al., 2011; Macnab et al., 1989). Due to the nonspecific nature of these symptoms, Cannabis intoxication can be easily misdiagnosed, potentially leading to inappropriate interventions such as antibiotic therapy, unnecessary imaging, or invasive diagnostic procedures like lumbar puncture (Lavi et al., 2016). For this reason, toxicological screening is essential for accurate diagnosis and appropriate clinical management.

The two main components of Cannabis are 9-tetrahydrocannabinol (THC) and cannabidiol (CBD). Given the highly variable and unpredictable pharmacokinetics (PK) of THC and CBD, both in therapeutic contexts and intoxication, plasma level monitoring is strongly recommended (Cafaro et al., 2025; Pigliasco et al., 2024).

In this paper we describe the clinical presentation and corresponding plasma levels of THC and CBD following unintentional ingestion of CCPs in two pediatric patients.

## Case presentation

### Case 1

A 1-year-old female patient was transferred from a peripheral hospital due to drowsiness and a suspected critical episode. The episode was described as crying followed by stiffening and shaking of the limbs, lasting a few seconds. In the days leading up to admission, she had been generally well, with only a mild cough and rhinitis, and no fever or other significant symptoms. The patient’s parents denied any history of trauma or accidental drug ingestion. Upon physical examination, the patient weighed 10 kg, had a Glasgow Coma Scale (GCS) score of 8, a heart rate of 174 bpm, respiratory rate of 15 breaths/min, and oxygen saturation (SaO2) of 96%, Poisoning Severity Score (PSS) (Persson et al., 1998) of 3. She was unconscious, responding only to painful stimuli, with noted mydriasis, absent photomotor reflexes, and generalized hypotonia. Cardiothoracic and abdominal examinations were unremarkable, with no meningeal signs. Hydration status was normal, and capillary refill time was less than 2 s. Endorectal administration of 5 mg of diazepam (Micropam^®^, 5 mg/2.5 mL, rectal solution) did not provide any benefit. Laboratory tests, including blood chemistry and venous pH, were within normal limits. An urgent brain cranial computed tomography (CT) scan showed no abnormalities. Intravenous hydration was initiated with normal saline and 5% dextrose at 40 mL/h, later increased to 70 mL/h. Urine toxicology screening was positive for THC, with results confirmed by both the initial Quidel Triage TOX immunoassay (94600; Quidel Cardiovascular Inc., San Diego, United States) and subsequent LC-MS/MS analysis.

### Case 2

Male, 15 months old, transferred from a peripheral hospital due to a state of unconsciousness that arose after an afternoon nap, a doubtful critical episode treated with midazolam and levetiracetam without benefit. In the previous days, regular wellbeing. No trauma reported, accidental intake of drugs and/or other substances denied. On physical examination, the child weighed 12 kg, with a GCS score of 3, a heart rate of 100 bpm, a respiratory rate of 16 breaths/min, and a SaO2 of 98%, PSS of 3 (Persson et al., 1998). He was unconscious but responsive to painful stimuli, with fixed mydriasis, absent pupillary reflexes, trismus, and nuchal rigidity. The patient also presented with a flexed posture and more pronounced hyposthenia on the right side. Capillary refill time was less than 2 s, while cardiothoracic and abdominal examinations were normal. Initial investigations, including blood tests, brain CT, EEG, and ECG, showed no abnormalities. An immunometric urine toxicology screening conducted at the peripheral hospital, which focused solely on drugs of abuse, returned a positive result for benzodiazepines, consistent with the medications administered upon admission. The subsequent brain magnetic resonance imaging was also negative. Intravenous hydration with normal saline and 5% dextrose was initiated at 50 mL/h. The patient received ceftriaxone and acyclovir after consultation with an infectious disease specialist for suspected meningoencephalitis. The patient was transferred to the intensive care unit (ICU), where a lumbar puncture was performed to investigate suspected encephalitis, which returned negative results. A toxicological assessment was subsequently performed under chain of custody. The initial urine toxicology screening, conducted using the Quidel Triage TOX immunoassay (94600; Quidel Cardiovascular Inc., San Diego, United States), tested positive for THC. This finding was confirmed by a follow-up analysis using LC-MS/MS.

## Toxicokinetic analysis

**FIGURE 1:**
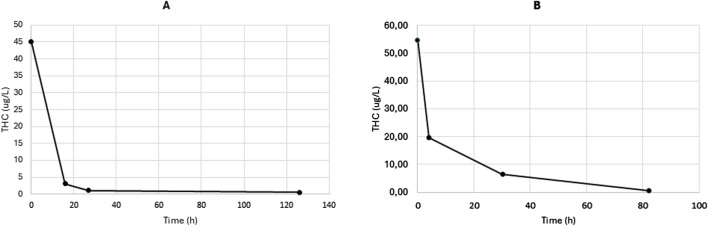
THC concentration vs. time profile in linear-linear scale for Case 1 **(A)** and Case 2 **(B)**.

Blood samples for TK analysis were collected by venipuncture into K_3_EDTA tubes (four samples at random time per patient). Plasma was separated by centrifugation at 4,000 *g* for 5 min and stored at −20 °C until analysis. Quantification of THC and CBD plasma concentrations was performed at the Giannina Gaslini Institute (Genoa, Italy) using a previously validated liquid chromatography–tandem mass spectrometry (LC-MS/MS) method, as described by our group (Barco et al., 2018). The 8-point calibration curve ranged from 0.2 to 300 μg/L for both THC and CBD. The THC concentration–time profiles for both patients are shown in Figure 1 (panel A: Case 1; panel B: Case 2). In Case 1, the ingestion of CCP was estimated to have occurred approximately 7 h prior to the first blood sample (THC concentration: 45.0 μg/L). In Case 2, ingestion was presumed to have occurred approximately 10 h before the first sample was drawn (THC concentration: 54.7 μg/L). CBD was undetectable in all samples from Case 1, while it was measurable only in the first plasma sample from Case 2 (CBD concentration: 1.11 μg/L).

TK parameters were determined using non-compartmental analysis (NCA) of the concentration–time data, performed with Phoenix WinNonlin Professional Edition v8.4 (Certara, France Sarl). The elimination rate constant (k_el_) of THC was calculated by log-linear regression of the terminal phase of the plasma concentration–time curve. Because the exact time of ingestion was unknown in both cases, the first concentration point was considered as time zero and excluded from the regression analysis. The estimated THC k_el_ values were 0.013 h^−1^ for Case 1 and 0.031 h^−1^ for Case 2, corresponding to terminal half-lives (t½) of 52.5 and 21.7 h, respectively. Plasma concentrations and toxicokinetic parameters of Δ^9^-THC and CBD in the two cases are summarized in Table 1.

**TABLE 1:** Plasma concentrations and toxicokinetic parameters of Δ^9^-THC and CBD in the two pediatric intoxication cases.

	Sampling time* (h after hospital admission)	THC (µg/L)	CBD (µg/L)
Case 1			
Sample 1	0	45	<0.2
Sample 2	16	3.1	<0.2
Sample 3	27	1.1	<0.2
Sample 4	126	0.5	<0.2
Elimination rate constant (kel) (h^−1^)	0.013		
Terminal half-life (t½) (h)	52.5		
Case 2			
Sample 1	0	54.67	1.11
Sample 2	4	19.76	<0.2
Sample 3	30	6.54	<0.2
Sample 4	82	0.71	<0.2
Elimination rate constant (kel) (h^−1^)	0.031		
Terminal half-life (t½) (h)	21.7		

*Exact time of ingestion unknown; the first blood sample (time = 0) was considered as reference for toxicokinetic analysis.

## Discussion

The two cases presented highlight the importance of considering Cannabis intoxication in the differential diagnosis of infants presenting with unexplained neurological symptoms, particularly in the absence of fever or signs of infection. As Cannabis use becomes increasingly widespread and accidental pediatric exposures rise, emergency physicians and pediatricians must remain vigilant in recognizing the potential for THC intoxication in young children.

Both infants in our cases exhibited central nervous system depression, a hallmark feature of Cannabis intoxication, yet their initial workups included considerations of infectious and metabolic causes. This underscores a critical issue: the potential for unnecessary and invasive investigations, such as lumbar punctures, CT scans, or empirical antiviral and antibiotic therapies, when a toxicological cause is overlooked. These interventions not only increase healthcare costs and resource utilization but also expose pediatric patients to unnecessary risks, including radiation exposure and medication side effects.

Routine toxicology screening should be strongly considered in infants and young children presenting with altered mental status, hypotonia, or excessive sleepiness in the absence of fever or other clear signs of infection, as it can prevent unnecessary interventions and facilitate timely and appropriate clinical management.

Rapid urine drug screening represents a non-invasive, cost-effective, and timely diagnostic tool that can prevent unnecessary investigations and accelerate appropriate clinical management. A positive toxicology result facilitates early diagnosis, supports the prompt initiation of supportive care, and provides reassurance to families. It also enables the activation of targeted social interventions to prevent future exposures.

Importantly, a positive screening result should be viewed as a clinical “red flag” warranting more specific and accurate confirmatory testing, such as second-tier toxicological analysis and, when available, therapeutic drug monitoring (TDM) using LC-MS/MS. This stepwise diagnostic strategy ensures precise identification of the substance involved, more accurate assessment of the degree of exposure, and ultimately more informed and effective clinical decision-making.

In our two pediatric intoxication cases, the observed plasma peak concentrations (45 μg/L and 54.7 μg/L, respectively) were markedly higher than the mean Cmax values previously reported in pediatric patients treated with standardized cannabis preparations for refractory epilepsy, analyzed using the same LC–MS/MS methodology (Gherzi et al., 2020). In that cohort, administered THC doses ranged from 6.6 mg to 33.0 mg, yielding proportionally lower plasma concentrations. A comparable dose–Cmax relationship has been reported in adults by Pellesi et al. (2018), who observed mean Cmax values of approximately 3 μg/L following ingestion of 50 mg THC edibles, consistent with the findings of Vandrey et al. (2017).

Conversely, the peak THC concentrations observed in our two cases are consistent with those described by Guidet et al. (2020), who reported plasma THC levels ranging from 4.4 to 127 ng/mL in 10 infants admitted for accidental cannabis intoxication. These similarities further support the clinical plausibility of our toxicokinetic findings and underline the marked variability in systemic THC exposure following unintentional ingestion in young children.

With regard to elimination kinetics, the estimated half-lives (52.5 h and 21.7 h) indicate markedly prolonged THC persistence compared with adult data, where half-lives typically range from 20 to 30 h. When compared with both our pediatric reference data and available adult studies, this prolongation may suggest a dose-dependent kinetic behavior in acute intoxication settings.

Due to limited information on the time interval between ingestion and blood collection, it was not possible to reliably assess the relationship between plasma THC concentrations and the severity of clinical manifestations. However, all subsequent blood samples were collected at defined intervals relative to the patients’ arrival at the hospital, providing a consistent temporal framework for toxicokinetic interpretation.

It should be noted that urine THC concentrations primarily reflect prior exposure and do not predict current impairment (Fitzgerald et al., 2023). Similarly, while the toxicokinetic data obtained in these pediatric cases provide valuable information on the timing and extent of exposure, they cannot be directly translated into an assessment of functional impairment. The toxicokinetic evaluation is further limited by the small number of samples (four per patient) and the inclusion of only two cases, which precludes broader generalization of the findings.

In addition to clinical and toxicokinetic considerations, preventive measures such as caregiver education and safer drug packaging may contribute to reducing the risk of accidental intoxications and to guiding appropriate social and child protection interventions (Amirshahi et al., 2019; Zwiebel et al., 2025). In conclusion, THC intoxication should be included in the differential diagnosis of any infant presenting with unexplained neurological symptoms, particularly in afebrile cases. Implementing early toxicological screening in such scenarios can prevent unnecessary invasive procedures, reduce the misuse of antimicrobials, and improve patient outcomes through appropriate and timely diagnosis.

## Data Availability

The original contributions presented in the study are included in the article/supplementary material, further inquiries can be directed to the corresponding author.
